# CARD8: A Novel Inflammasome Sensor with Well-Known Anti-Inflammatory and Anti-Apoptotic Activity

**DOI:** 10.3390/cells13121032

**Published:** 2024-06-13

**Authors:** Tugay Karakaya, Marta Slaufova, Michela Di Filippo, Paulina Hennig, Thomas Kündig, Hans-Dietmar Beer

**Affiliations:** 1 Department of Dermatology, University Hospital Zurich, CH-8952 Schlieren, Switzerland; tugay.karakaya@usz.ch (T.K.); marta.slaufova@usz.ch (M.S.); michela.difilippo@usz.ch (M.D.F.); paulina.hennig@usz.ch (P.H.); thomas.kuendig@usz.ch (T.K.); 2 Faculty of Medicine, University of Zurich, CH-8006 Zurich, Switzerland

**Keywords:** CARD8, NLRP1, NLRP3, inflammasome, pyroptosis, inflammation

## Abstract

Inflammasomes comprise a group of protein complexes with fundamental roles in the induction of inflammation. Upon sensing stress factors, their assembly induces the activation and release of the pro-inflammatory cytokines interleukin (IL)-1β and -18 and a lytic type of cell death, termed pyroptosis. Recently, CARD8 has joined the group of inflammasome sensors. The carboxy-terminal part of CARD8, consisting of a function-to-find-domain (FIIND) and a caspase activation and recruitment domain (CARD), resembles that of NLR family pyrin domain containing 1 (NLRP1), which is recognized as the main inflammasome sensor in human keratinocytes. The interaction with dipeptidyl peptidases 8 and 9 (DPP8/9) represents an activation checkpoint for both sensors. CARD8 and NLRP1 are activated by viral protease activity targeting their amino-terminal region. However, CARD8 also has some unique features compared to the established inflammasome sensors. Activation of CARD8 occurs independently of the inflammasome adaptor protein apoptosis-associated speck-like protein containing a CARD (ASC), leading mainly to pyroptosis rather than the activation and secretion of pro-inflammatory cytokines. CARD8 was also shown to have anti-inflammatory and anti-apoptotic activity. It interacts with, and inhibits, several proteins involved in inflammation and cell death, such as the inflammasome sensor NLRP3, CARD-containing proteins caspase-1 and -9, nucleotide-binding oligomerization domain containing 2 (NOD2), or nuclear factor kappa B (NF-κB). Single nucleotide polymorphisms (SNPs) of *CARD8*, some of them occurring at high frequencies, are associated with various inflammatory diseases. The molecular mechanisms underlying the different pro- and anti-inflammatory activities of CARD8 are incompletely understood. Alternative splicing leads to the generation of multiple CARD8 protein isoforms. Although the functional properties of these isoforms are poorly characterized, there is evidence that suggests isoform-specific roles. The characterization of the functions of these isoforms, together with their cell- and disease-specific expression, might be the key to a better understanding of CARD8’s different roles in inflammation and inflammatory diseases.

## 1. The Caspase Activation and Recruitment Domain

The caspase activation and recruitment domain (CARD) is commonly found in proteins associated with inflammation and regulated cell death pathways [1,2]. Together with the death effector domain (DED), pyrin domain (PYD), and death domain (DD), they comprise a highly conserved structure consisting of six to seven alpha helices, termed death domain fold, allowing homotypic (e.g., CARD-CARD), but never heterotypic (e.g., PYD-CARD), protein-protein interactions [3]. These homotypic interactions can induce the assembly of large protein complexes, like inflammasomes or the apoptosome. The cysteine protease caspase-1 is known as the prototypic CARD-protein, which plays a fundamental role in the induction of inflammatory responses [4].

## 2. Caspase-1 and Inflammasomes

Caspase-1 cleaves, and thereby activates, the pro-inflammatory cytokines interleukin (IL)-1β and -18. IL-1β is a potent cytokine, capable of initiating an inflammatory response through the recruitment and activation of neutrophils and other immune cells. While it is essential for immunity and repair processes, its activity also contributes to the pathogenesis of numerous inflammatory diseases [5,6,7]. Consequently, several drugs antagonizing IL-1β activity were approved for the treatment of patients suffering from IL-1-associated inflammatory diseases [8,9,10]. IL-18 induces expression of interferon gamma (IFN-γ) and activation of T cells and natural killer (NK) cells [11]. IL-1β and -18 lack a signal peptide for secretion through the canonical endoplasmic reticulum/Golgi-dependent pathway. These cytokines are released through holes in the cytoplasmic membrane, formed by proteins of the gasdermin family [12]. Caspase-1 cleaves and activates gasdermin D (GSDMD), leading to the formation of pores in the plasma membrane, and allowing for the release of IL-1β and -18 [13]. Furthermore, GSDMD activation can induce a lytic and highly inflammatory type of regulated cell death, termed pyroptosis [14].

Caspase-1 is initially expressed in an enzymatically inactive pro-form, which is activated through proximity-induced self-processing, induced by the assembly of inflammasomes [15,16]. These multi-protein complexes consist of a sensor protein, such as NLR family pyrin domain containing 1 (NLRP1) [17], NLRP3 [18], NLR family CARD domain containing protein 4 (NLRC4) [19], absent in melanoma 2 (AIM2) [20], or Pyrin [21], the adaptor protein ASC, and the effector protein caspase-1. The sensors detect certain exogenous or endogenous stress factors, termed pathogen-associated molecular patterns (PAMPs) or damage/danger-associated molecular patterns (DAMPs), which induces oligomerization of the sensors and recruitment of ASC, leading to the formation of ASC polymers called ASC-specks, and subsequent caspase-1 recruitment and activation. The assembly of inflammasomes is mediated by homotypic interactions of CARDs (in the case of NLRP1, NLRC4, ASC, and caspase-1) and PYDs (in the case of NLRP3, AIM2, Pyrin, and ASC). The most extensively studied inflammasome sensor is NLRP3 [16]. Despite its lesser importance for general immunity, NLRP3 plays a crucial role in major inflammatory diseases, such as atherosclerosis or Alzheimer’s disease. Therefore, its pharmacological targeting represents a promising strategy for treating patients with NLRP3-associated conditions, with minimal impact on their overall immune system [22]. Inflammasome proteins are particularly expressed by innate immune cells, such as macrophages and dendritic cells (DCs), but also by endothelial and epithelial cells [6]. Immune cells usually do not constitutively express IL-1β and certain other inflammasome proteins. Their expression is induced by bacterial lipopolysaccharides (LPS), double-stranded RNA (dsRNA), IFN-γ, tumor necrosis factor alpha (TNF-α), or IL-1 itself [23,24,25]. The induction of IL-1β expression is mainly mediated by the activation of the transcription factor nuclear factor kappa B (NF-κB) [26].

## 3. The NLRP1 Inflammasome

NLRP1 and CARD8 are the only known proteins containing a function-to-find domain (FIIND). Full-length NLRP1 is a 1473 amino acid-long protein, consisting of an amino-terminal PYD, followed by a NACHT domain, six consecutive leucine-rich repeats (LRRs), a FIIND, and a carboxy-terminal CARD. Furthermore, several other isoforms with unknown biological functions arise from alternative splicing. For example, a completely inactive NLRP1 isoform lacks coding exon 14, resulting in a truncated FIIND [27,28]. NLRP1 is ubiquitously expressed in humans, with particularly high levels found in various immune cells, neurons, and keratinocytes [29]. The NLRP1 inflammasome pathway is poorly conserved between humans and rodents. For example, mice express three paralogues (Nlrp1a/b/c), all lacking the PYD [30]. Unlike other inflammasome sensors, the PYD of human NLRP1 represents an inhibitory domain, rather than an effector domain [29]. The FIIND of NLRP1 is a 286 amino acid-long domain, consisting of two subdomains, called ZU5 and UPA, with a SerPhe/Ser cleavage motif located in between [31,32]. It is believed that the FIIND is an autoproteolytic self-activation domain, with Ser1213 representing the active site for cleavage after Phe1212. However, the serine protease-properties of NLRP1 are poorly characterized. FIIND-mediated autocatalytic self-processing occurs in a constitutive manner and is necessary, but not sufficient, for NLRP1 activation, since the 1212 amino acid-long amino-terminal inhibitory fragment (N-NLRP1) remains non-covalently bound to the 261 amino acid-long carboxy-terminal effector fragment (C-NLRP1), inhibiting its activity [32]. Experiments in the murine system revealed that Nlrp1b activation requires ubiquitinylation and the subsequent proteasomal degradation of its amino-terminal fragment for activation [33]. The pathogen *Bacillus anthracis*, which is sensed by murine Nlrp1b, but not human NLRP1, expresses a protease, named anthrax lethal factor, which targets the amino-terminal fragment of Nlrp1b. Cleavage in the amino-terminus by this protease leads to the generation of a neo-amino-terminus, destabilizing N-Nlrp1b and leading to its degradation by the N-end rule pathway through its ubiquitinylation and subsequent proteasomal degradation [34,35,36]. Consequently, the carboxy-terminal effector fragment is no longer inhibited and can induce ASC-speck formation, followed by activation of caspase-1.

Human NLRP1 is activated through mechanisms other than murine Nlrp1b, upstream of proteasomal degradation of N-NLRP1. In 2007, the NLRP1 inflammasome was identified in human primary keratinocytes with UVB radiation as its activating stimulus [37,38], suggesting that NLRP1 activation in epidermal keratinocytes induces sunburn in humans [39]. Recently, it was demonstrated that UVB-induced NLRP1 activation in human keratinocytes is regulated through the ribotoxic stress response (RSR) via phosphorylation of N-NLRP1 by sterile alpha motif and leucine zipper containing kinase AZK alpha (ZAKα) and mitogen-activated protein kinase p38 (p38) [40,41]. In addition, the pore-forming bacterial toxin nigericin can induce N-NLRP1 phosphorylation and activation through the induction of ZAKα, p38, and c-Jun N-terminal kinase (JNK) activation [42]. Furthermore, long dsRNA, originating from viral infections, activates NLRP1 through direct binding [43]. However, also here, the RSR, leading to the phosphorylation of NLRP1, might as well contribute to the activation of the sensor [41]. Interestingly, NLRP1 senses viral infection also through a second mechanism. NLRP1 can act as a “tripwire” against certain viruses upon infection of human keratinocytes or other epithelial cells, which is activated after proteolytic cleavage in N-NLRP1 by viral 3C proteases [44,45,46]. Finally, NLRP1 is activated by Val-boroPro (VbP), also called talabostat, and other inhibitors of dipeptidyl peptidases 8 and 9 (DPP8/9) [47]. DPP8/9 represent a checkpoint for NLRP1 activation, as well as for Nlrp1b [48]. Cryogenic electron microscopy-derived structures revealed that a single DPP9 protein has the capacity to bind one N-NLRP1 and two C-NLRP1 molecules [49,50]. The amino-terminus of C-NLRP1 represents a DPP8/9 substrate sequence capable of binding to the peptidase’s active site without undergoing processing. Consequently, NLRP1 probably exists in a partially activated state, even during homeostasis, due to degradation of N-NLRP1. This activation is likely restrained by its interaction with DPP8/9.

NLRP1 is the central inflammasome sensor in human keratinocytes and the skin [51,52]. Gain-of-function mutations of *NLRP1* cause inflammatory skin syndromes and predispose patients to the development of squamous cell carcinomas (SCCs), a major type of skin cancer [29]. In addition, SNPs of *NLRP1* are associated with several inflammatory diseases with a predominant skin phenotype, emphasizing the central role of NLRP1 in keratinocytes and the human skin [27,28].

## 4. CARD8: An Introduction

**Figure 1:**
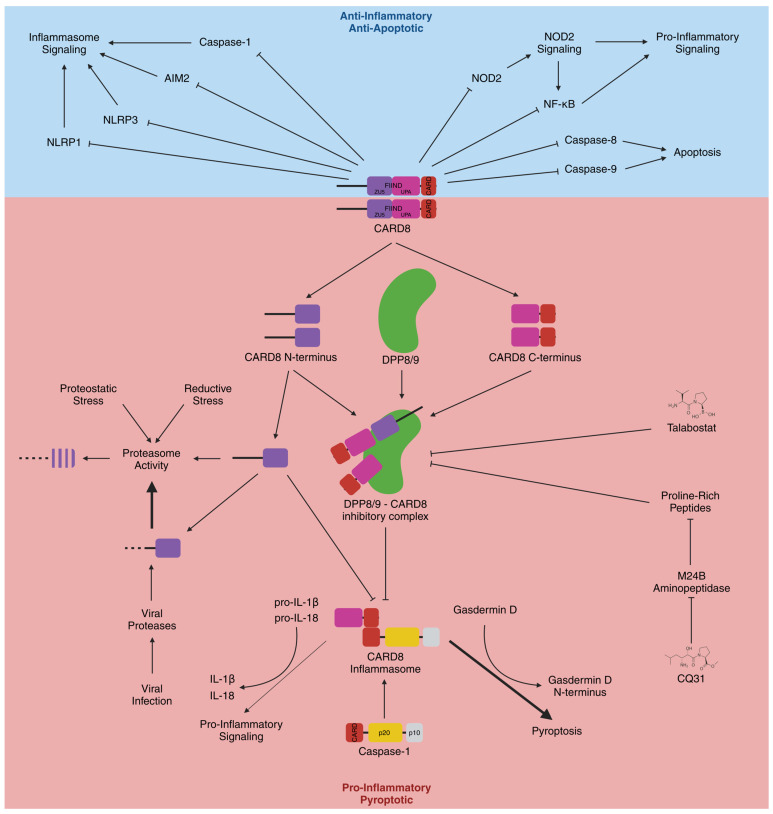
**CARD8 has anti- and pro-inflammatory activity.** CARD8 can directly interact with NLRP1, NLRP3, and caspase-1, and inhibit the activation of the NLRP1, NLRP3, and AIM2 inflammasomes. CARD8 can negatively regulate NOD2, NF-κB, and apoptosis, the latter through an indirect or direct inhibition of caspase-8 or -9, respectively. Moreover, after autoproteolytic self-activation, CARD8 forms an ASC-independent inflammasome, activated by inhibition of the dipeptidyl peptidases DPP8/9, by talabostat or CQ31, or by enhanced proteasomal degradation of the inhibitory amino-terminus, mediated through proteolytic processing of its amino-terminal disordered region by viral proteases and potentiated by increased proteasome activity. Caspase-1 recruitment and activation by autocatalytic cleavage and release of the subunits p20 and p10 through the CARD8 inflammasome is mainly associated with pyroptosis.

**Figure 2:**
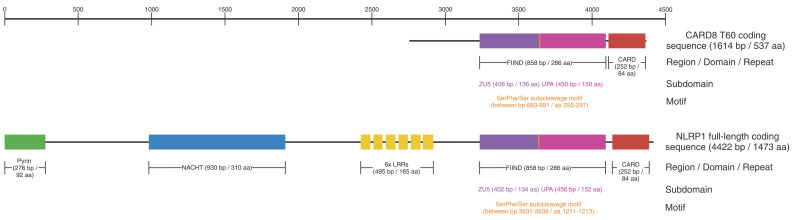
**CARD8 and NLRP1 show high structural homology.** Side-by-side comparison of the longest isoforms of CARD8 and NLRP1 highlights the high structural similarities between the two proteins, especially regarding the FIIND and CARD.

**Figure 3:**
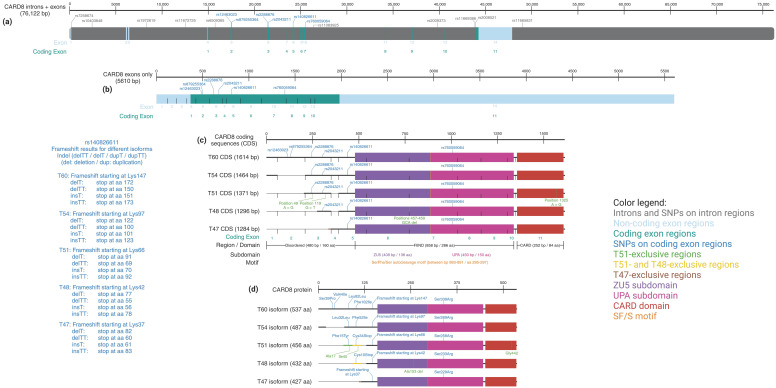
**The *CARD8* gene, its most important SNPs, and protein isoforms.** (**a**) The *CARD8* gene locus, including introns, exons, and localization of selected disease-associated SNPs. (**b**) *CARD8* coding and non-coding exons and their SNPs. (**c**) *CARD8* coding sequences of all reported isoforms, their domains, and SNPs. (**d**) CARD8 protein sequences of all reported isoforms and consequences of the SNPs on the protein level. Results of the indel mutation rs140826611 on the protein level and color code of the Figure are shown on the left and right, respectively [168,169,170].

More than 20 years ago, several groups identified CARD8 as a 48 kDa protein, also termed CARD-inhibitor of NF-kappa-B-activating ligand (CARDINAL) or tumor-up-regulated CARD-containing antagonist of caspase 9 (TUCAN) (Figure 1) [53,54,55]. CARD8 possesses a disordered amino-terminus, but its carboxy-terminal part, with its FIIND and CARD, resembles that of NLRP1 (Figure 2). CARD8 is reported to be ubiquitously expressed in different human tissues and cell types, and its expression is particularly upregulated in different types of cancer [53,56]. In contrast to NLRP1 and all other known inflammasome sensors, CARD8 does not have orthologues in rodents [57]. Via its CARD, CARD8 interacts with itself [53,58], but also binds and inhibits caspase-9, suggesting that it plays a role as an anti-apoptotic protein [53]. Furthermore, CARD8 binds and inhibits caspase-1, thereby acting as a negative regulator of IL-1β activation [54]. The role of the 48 kDa CARD8 protein, also called CARD8 T48, in apoptosis is still under debate [54,55]. Moreover, by binding to nuclear factor kappa B essential modulator (NEMO), CARD8 T48 inhibits NF-κB activation, which is required for the induction of the expression of inflammasome proteins, particularly IL-1β [26,54,55]. In addition to caspase-1, CARD8 also interacts with the NACHT domain of NLRP3 and was identified as a component of the NLRP3 inflammasome [59]. Later, a novel 54 kDa isoform of CARD8 with an extended amino-terminus, called CARD8 T54, was described (Figure 3). This isoform is highly expressed in different human cancers [60]. Via its interaction with the Fas-associated death domain protein (FAAD), CARD8 T54 can inhibit the activation of another pro-apoptotic caspase, caspase-8 [60]. Later, three more isoforms, called CARD8 T60, T51, and T47, were identified [61]. Tissue- and cell-type-specific expression of CARD8 isoforms, and especially their functional properties, are still poorly characterized.

## 5. The CARD8 Inflammasome

The CARD8 T60 isoform was identified as a sensor for HIV protease activity in HIV-1-infected CD4^+^ T cells and macrophages [62]. Pathogen sensing by CARD8 resulted in pyroptosis of infected cells and IL-1β secretion in macrophages, THP-1 cells, or transfected HEK293 cells [62]. In patients, HIV-1 persists even after anti-retroviral therapy in CD4^+^ T cells [63]. However, some anti-HIV drugs, which inhibit reverse transcriptase activity, induce HIV-protease activation in macrophages and CD4^+^ T cells [62,63]. Thereafter, CARD8 T60 can be cleaved in the amino-terminal disordered region, causing the proteasomal degradation of the amino-terminal inhibitory fragment, followed by direct caspase-1 recruitment and activation, and eventually pyroptosis of the infected cells. This shows that CARD8 can form an inflammasome, but in contrast to other human inflammasome sensors, without requiring the adaptor protein ASC [62]. Interestingly, HIV-1 protease cleaves CARD8 T60 at Phe59, a location that is missing in T54, T51, T48, and T47. A few years before that, talabostat was shown to activate CARD8 T60 in an ASC-independent manner in THP-1 and several other acute myeloid leukemia (AML) cells, inducing pyroptosis, but barely any cytokine secretion [64]. The ability to induce CARD8-dependent pyroptosis in AML and other cells correlated roughly with the protein expression level of CARD8 [64].

Structural differences between the CARDs of NLRP1 and CARD8 might be the reason why NLRP1 requires ASC for subsequent caspase-1 recruitment, whereas CARD8 can directly interact with the protease [65,66,67]. Furthermore, unlike NLRP1, CARD8 binds to DPP9 outside of its catalytic pocket [66]. In contrast to talabostat, CQ31 is a selective CARD8 activator, inducing a weaker inhibition of DPP8/9 sufficient for release and activation of CARD8, but not NLRP1 [68,69,70]. Nevertheless, DPP8/9 represent a checkpoint for both inflammasome sensors. As in AML cells, talabostat induces CARD8 T60-dependent and ASC-independent pyroptosis without cytokine release in resting T cells [71,72]. However, upon T cell receptor-activation and differentiation, T cells lose the ability to activate CARD8. While the exact mechanism underlying this regulation is unknown, it might be related to the reduced protein expression of CARD8 upon differentiation of T cells [71,72]. As talabostat also induces pyroptosis in HIV-1-infected cells, the DPP8/9 inhibitor can be used to support the elimination of residual HIV-1-infected CD4^+^ T cells via CARD8 T60-induced pyroptosis [73,74]. However, as suggested by experiments with T cell receptor-activated and talabostat-treated T cells [71,72], T cell activation abolishes CARD8 function and increases permissiveness to infection [75]. Interestingly, only CARD8 from humans, but not from primates, contains the Phe59Phe60 motif in the amino-terminus of CARD8 T60 for proteolytic activation by HIV-1 protease, suggesting that this motif emerged after the evolutionary separation of chimpanzees and humans [76]. Through a similar mechanism, 3CL proteases derived from coronaviruses, including severe acute respiratory syndrome corona virus 2 (SARS-CoV-2), and human rhinovirus (HRV), are able to activate CARD8 in HEK293 and THP-1 cells [77,78]. The enterovirus coxsackie virus B3 (CVB3), that can induce myocardial injury by infecting aortic endothelial cells and cardiomyocytes, can also activate CARD8 [79,80]. The CVB3-encoded proteases 2A and 3C cleave CARD8 T60 at Gly38, causing proteasome-dependent CARD8 activation, followed by caspase-1 activation, pyroptosis, and IL-18 secretion. However, CARD8 activation is not protective in this case, but rather potentiates CVB3 replication and propagation [79]. Endothelial cells also express NLRP1, which is not required for CVB3-induced pyroptosis, but contributes to talabostat-induced pyroptosis. This raises the questions of whether and how CARD8 and NLRP1 can interact and crosstalk in different cell types [68,80]. Treatment of the human keratinocyte cell line N/TERT-1 with talabostat induces pyroptosis and the secretion of high levels of IL-1β, which is NLRP1- but not CARD8-dependent, despite high expression of both sensors [47]. However, when CARD8 T60 is overexpressed in NLRP1 knockout N/TERT-1 keratinocytes, talabostat-induced IL-1β secretion can be rescued [43]. The molecular mechanisms underlying these effects are unknown to date.

Certainly, CARD8 and NLRP1 have a lot in common [57,67]. Both can be activated by proteostatic stress initiated through DPP8/9 inhibition, inducing functional degradation of their amino-terminal inhibitory fragments [81]. Furthermore, protein-folding stress, for example as a consequence of chaperone inhibition or treatment of cells with initiators of the unfolded protein response, is not sufficient, but significantly potentiates NLRP1 or CARD8 inflammasome activation [82]. This is also the case for reductive stress induced by, for example, treatment with ferroptosis inhibitors that support the degradation of the amino-terminal fragments of NLRP1 and CARD8, and thereby potentiate inflammasome activation [83]. In contrast to N-NLRP1, which is believed to be ubiquitinylated and degraded by the proteasome [34,35], the amino-terminal fragment of CARD8 is degraded by the 20S proteasome without the need for its previous ubiquitinylation [84].

In conclusion, at least CARD8 T60 is known to form an inflammasome similar to NLRP1. However, in contrast to NLRP1, CARD8 can recruit and activate caspase-1 directly, without requiring ASC. Although it is believed that CARD8 activation primarily induces pyroptosis, this might be dependent on the employed cell types, treatment conditions, or experimental models, since CARD8 activation can be associated with IL-1β and IL-18 secretion under certain conditions [62,79]. It is not known whether CARD8 T60 is the only isoform able to form an inflammasome. However, this is strongly suggested due to the fact that several of its activators require the full-length amino-terminal disordered region only present in CARD8 T60 [85].

## 6. CARD8 Inhibits Inflammasome Activation and Pro-Inflammatory Signaling

Aberrant NLRP3 activation underlies many common inflammatory diseases [6,22]. Therefore, the scientific and medical interest in the molecular mechanisms underlying NLRP3 activation is very high. When the NLRP3 inflammasome was described for the first time, CARD8 was shown to be an integral component of the complex [59]. Immunoprecipitation experiments demonstrated an interaction between CARD8 T48 and NLRP3 mediated by the FIIND of CARD8 and the NACHT domain of NLRP3 [59]. Overexpression and knockdown experiments revealed that CARD8 T48 can inhibit NLRP3 inflammasome activation [86]. Gain-of-function mutants of *NLRP3*, which are the cause of the auto-inflammatory disorder cryopyrin-associated periodic syndrome (CAPS) [87], cannot interact with CARD8, suggesting that the lack of NLRP3 inhibition by CARD8 is the reason for the inflammatory phenotype in CAPS patients [86]. The *CARD8* variant rs140826611 is associated with periodic fever with aphthous stomatitis, pharyngitis, and cervical adenitis (PFAPA), an auto-inflammatory disease affecting mainly children (Table 1) [88]. This leads to an indel mutation, resulting in a frameshift and loss of the FIIND and CARD. Consequently, this CARD8 mutant cannot bind and inhibit NLRP3, suggesting that the resulting increase in NLRP3 activation underlies the inflammatory phenotype in children suffering from PFAPA [88]. Another *CARD8* SNP, called rs879255364, is associated with Crohn’s disease (CD), one of the two main forms of inflammatory bowel disease (IBD) [89]. While this mutation is located upstream of the start codon of CARD8 T48, it causes a missense mutation (Val44Ile) in CARD8 T60. CARD8 T60 was shown to inhibit NLRP3 activation comparably to T48, and the Val44Ile mutant bound and inhibited NLRP3 to a lesser degree than wild-type CARD8 T60 [89]. Most importantly, overexpression experiments demonstrated that this mutant CARD8 T60 variant had a dominant-negative effect on CARD8 T48 and T60 concerning the inhibition of NLRP3 inflammasome activation [89]. Therefore, in cells of these patients suffering from CD, the CARD8 T60 Val44Ile mutant likely abrogates inhibition of NLRP3 by CARD8 T48 and wild-type T60 through hetero-oligomerization [89]. An important question here is, however, how a mutated amino-terminus in CARD8 T60 Val44Ile can prevent binding of wild-type T60 and T48 to NLRP3, if the interaction is mediated by the FIIND [59], which is believed to be constitutively processed [32]. DCs of CD patients with rs879255364 are characterized by stronger AIM2, but not Pyrin or NLRC4 inflammasome activation, suggesting that CARD8 also inhibits AIM2 activation, although the molecular mechanisms underlying this inhibition are not known yet [89].

Overexpression experiments in HEK293 cells, reconstituted with NLRP1 inflammasome components, demonstrated that CARD8 T60, but not CARD8 T48, binds and inhibits NLRP1 activation [90]. Expression of T60 in human skin was confirmed at the transcriptional level. Co-immunoprecipitation experiments revealed that the ZU5 part of the FIIND of CARD8 can interact with the NACHT, LRRs, and FIIND of NLRP1 [90]. Interestingly, CARD8 T60 was found to inhibit wild-type NLRP1, but not two gain-of-function mutants of NLRP1, suggesting that these mutants may support NLRP1 activation, as their interaction with CARD8 is negatively affected [90]. However, it should be considered that this interpretation is based on less physiological overexpression experiments, where the autocatalytic self-processing of CARD8 did not occur [89,90]. Nevertheless, experiments in endothelial cells and keratinocytes, which express both NLRP1 and CARD8 endogenously, also suggest a crosstalk of NLRP1 and CARD8 [43,79,80].

Caspase-1 is the effector protein of all inflammasomes [16]. CARD8 T48 interacts with caspase-1, as well as with the caspase-1-inhibiting CARD-only proteins (COPs) ICEBERG and pseudo-ICE, which is believed to be mediated through homotypic CARD-CARD interactions [54,59]. CARD8 inhibits caspase-1 activity and reduces IL-1β secretion when overexpressed in HEK293 or THP-1 cells [54,59]. Whether CARD8 is a general inhibitor of inflammasome activation through its interaction with caspase-1 or mainly antagonizes certain inflammasome sensors through direct interactions, particularly NLRP3, is not known.

The transcription factor NF-κB is a central regulator of inflammasomes, since it regulates the expression of certain inflammasome components [23,24,25]. CARD8 T48 inhibits NF-κB through multiple pathways, including an interaction with NEMO [54,55,58]. In intestinal epithelial cells, CARD8 T48 has an inhibitory effect on nucleotide-binding oligomerization domain containing 2 (NOD2). NOD2 is an intracellular pattern-recognition receptor with an important role in CD that can regulate NF-κB activation through direct interaction, and is inhibited by the interaction between the FIIND of CARD8 T48 and the NACHT domain of NOD2 [91].

In summary, CARD8 exhibits the ability to counteract the inflammasome pathway at various stages, notwithstanding the fact that CARD8 T60 has the capability to independently form a functional inflammasome as well. However, it is not known whether some of these effects of CARD8 are limited to certain cell types or experimental conditions. Despite existing evidence for isoform-specific activities of CARD8 [90], their properties, especially regarding the crosstalk of CARD8 isoforms with each other, are poorly understood [89].

**Table 1:** **Selected SNPs located on the genomic locus of *CARD8*.** Selected *CARD8* SNPs located within the genomic region of the gene, with their exact positions, variation types (single nucleotide variants or insertions/deletions), alleles, genetic consequences, reported phenotypes, and references.

Reference SNP	Position	Var. Type	Alleles	Genetic Consequence	Reported Phenotypes
**rs7258674**	chr19:48255771 (GRCh38.p14)	SNV	A>G	Intron Variant	-Increased glucose levels [92].
**rs10403848**	chr19:48253518 (GRCh38.p14)	SNV	G>A/ G>T	Intron Variant	-Increased risk of psoriasis vulgaris [93].
**rs1972619**	chr19:48247672 (GRCh38.p14)	SNV	C>T	Intron Variant	-Associated with the pathogenesis of Crohn’s disease [94].
**rs11672725**	chr19:48243424 (GRCh38.p14)	SNV	C>G/ C>T	Intron Variant	-Increased risk of adult-onset Still’s disease [95]. -Increased risk of gastric cancer [96]. -Increased risk of ischemic stroke combined with NLRP3 rs10754558 [97].
**rs6509365**	chr19:48240212 (GRCh38.p14)	SNV	A>G	Intron Variant	-Influences IL-1β, IL-6, and IL-33 plasma levels [98]. -Protective against sporadic malignant melanoma [99]. -Increased susceptibility to *Mycobacterium tuberculosis* infection in HIV-positive patients [63,100].
**rs12463023**	chr19:48238477 (GRCh38.p14)	SNV	A>G	Missense Variant	-Higher specificity for certain picornavirus proteases [45].
**rs879255364**	chr19:48238462 (GRCh38.p14)	SNV	C>T	Missense Variant	-Higher IL-1β levels, and supports Crohn’s disease pathogenesis [89].
**rs2288876**	chr19:48234507 (GRCh38.p14)	SNV	A>G/ A>T	Missense Variant	-Influences IL-8 levels and contributes to development of cutaneous leishmania caused by *Leishmania guyanensis* infection [101].
**rs2043211**	chr19:48234449 (GRCh38.p14)	SNV	A>T	Stop Gained	-Lower levels of CCL20 and IL-6 in healthy patients [102]. -Worse pathogenesis of rheumatoid arthritis with NLRP3 rs35829419 [103] or rs4612666 [104]. -Associated with rheumatoid arthritis [103,105,106,107,108,109], or no association [57,110,111]. -Protective against SARS-CoV-2 [112]. -Increased susceptibility to chronic kidney disease with or without NLRP3 rs10754558 [113]. -Increased susceptibility to psoriasis [114]. -Associated with inflammatory bowel disease [115,116], or no association [57,117,118,119]. -Ulcerative colitis [94]. -Crohn’s disease [120,121,122]. -Neutropenia in hematological malignancies under chemotherapy [123]. -Cardiovascular diseases [124]. -Chronic myeloid leukemia [125]. -Arteriosclerosis obliterans [126]. -Preeclampsia [127]. -Atopic dermatitis [128]. -Bacterial meningitis [129]. -Lower *Mycobacterium tuberculosis* susceptibility of macrophages in combination with NLRP3 rs35829419 [130]. -Higher risk of bacteraemia [131]. -Associated with extrapulmonary tuberculosis in combination with NLRP3 rs35829419 [132]. -Higher susceptibility to multiple myeloma [133]. -Higher plasma levels of IL-1β and IL-33 in combination with NLRP3 rs35829419 [134]. -Associated with psoriatic arthritis [135]. -Delayed apoptosis in neutrophils in combination with NLRP3 rs35829419 [136]. -No association with susceptibility to cardiovascular events and rheumatoid arthritis [137]. -Decreased risk of ankylosing spondylitis [138,139]. -Higher IL-1β levels [140]. -Increased risk of Alzheimer’s disease [141]. -Increased risk of Crohn’s disease and inflammatory bowel disease with or without NLRP3 rs35829419 [115,142,143]. -Increased risk of ischemic stroke with [144] or without NLRP3 rs35829419 [145]. -Increased risk of abdominal aortic aneurism in combination with NLRP3 rs35829419 [146]. -Increased susceptibility to B-Cell non-Hodgkin’s lymphoma development [147] -Higher risk of bone erosion in cholesteatoma patients [148]. -Higher IL-1β and IL-18 levels in combination with NLRP3 rs35829419 [149]. -Higher risk of large-artery atherosclerosis stroke [150]. -Higher risk of gout [105,151,152,153]. -Higher susceptibility to acute lymphoblastic leukemia [154].
**rs140826611**	chr19:48231761-48231763 (GRCh38.p14)	Indel	delTT/ delT/ dupT/ dupTT	Frame-shift Variant	-Increased risk of periodic fever with aphthous stomatitis, pharyngitis, and cervical adenitis [88], in combination with Kawasaki Syndrome [155].
**rs760059064**	chr19:48230456 (GRCh38.p14)	SNV	G>A/ G>T	Missense Variant	-Connected to juvenile idiopathic arthritis with associated inflammatory bowel disease [156].
**rs11083925**	chr19:48229560 (GRCh38.p14)	SNV	A>C/ A>T	Intron Variant	-Decreased risk of diabetic nephropathy [157].
**rs2009373**	chr19:48216157 (GRCh38.p14)	SNV	T>C/ T>G	Intron Variant	-Decreased risk of extrapulmonary tuberculosis development [158]. -Decreased risk of hepatitis C development [159].
**rs11669386**	chr19:48212089 (GRCh38.p14)	SNV	G>A/ G>T	Intron Variant	-Higher susceptibility to arterial hypertension [160,161].
**rs2008521**	chr19:48212012 (GRCh38.p14)	SNV	T>A/ T>C	Intron Variant	-Higher susceptibility to pneumococcal meningitis [162].
**rs11665831**	chr19:48206940 (GRCh38.p14)	SNV	T>A/ T>C/ T>G	Intron Variant	-Decreased risk of diabetic nephropathy [157].

## 7. SNPs of *CARD8* and *CARD8*-Associated Diseases

Several SNPs of *CARD8* have been identified that are associated with different inflammatory diseases (Table 1). The most prominent *CARD8* SNP is called rs2043211, where an A>T mutation in coding exon 3 leads to a nonsense mutation in the disordered amino-terminal region of CARD8 T48, therefore called C10X, and also in T51 (Cys34Stop). In contrast, it only results in the substitution of a single amino acid in CARD8 T60 (Phe102Ile) and T54 (Phe52Ile), while the T47 isoform is not affected [63]. Since T48 was initially identified as the canonical isoform of CARD8 [53,54,55], it was believed that homozygous carriers of rs2043211 do not express a functional CARD8 protein. Interestingly, with roughly 34% of heterozygous and 9% of homozygous carriers within certain populations, the frequency of rs2043211, and consequently the carriers of the homozygous “knockout” allele, is quite high [163,164]. These numbers might suggest that, in some populations, the lack of a functional CARD8 T48 might convey a certain evolutionary advantage. As previously discussed, CARD8 can inhibit NLRP3 activation and mutations of *CARD8*, which weaken this interaction, are connected to several NLRP3-associated diseases [59,86,88,89]. For example, NLRP3 activation underlies the pathophysiology of gout [18], and, indeed, the rs2043211 mutation was associated with this type of arthritis [105,151,152,153]. Several publications also claim a role for CARD8 in rheumatoid arthritis (RA) [103,105,106,107,108,165]. However, whether rs2043211 is indeed associated with RA is controversially discussed [57,110]. Similarly, there is evidence that carriers of rs2043211 have an increased risk for developing IBD [94,115]. However, this is also a matter of active debate [117,118,119]. Interestingly, carriers of rs2043211 can also be protected from certain diseases, such as non-Hodgkin’s B-cell lymphoma, psoriatic arthritis, or neutropenia after chemotherapy for hematological malignancies [123,135,147]. These contrasting effects might be reflective of the fact that different tissues and cell types might have varying expression patterns of certain CARD8 isoforms, some of which might have pro-inflammatory and others anti-inflammatory and anti-apoptotic activity. Furthermore, inflammation is not always detrimental, but is often required for tissue repair, which might therefore also be beneficial under certain conditions. CARD8 T60 senses HIV-1 by proteolytic processing in the amino-terminal part, which is missing in other isoforms, resulting in pyroptosis of infected cells, and supports the immune response against the infection [62]. Consequently, rs2043211, which negatively effects the expression of CARD8 T48, but not of T60, is not relevant for HIV-1 infection [63], whereas another intronic SNP, called rs6509365, contributes to *Mycobacterium tuberculosis* and HIV-1 co-infection [100]. The SNP rs12463023, leading to a Ser39Pro mutation in CARD8 T60, influences the cleavage by 3C^pro^ proteases of certain picornaviruses, which may again represent a case of evolutionary adaptation to combat virus infection [77]. The SNP rs35829419 (Glu705Lys) of *NLRP3*, which causes a gain-of-function mutation, is associated with the susceptibility to different autoimmune and inflammatory diseases, and occurs at a frequency of 7.2% [134,166]. Among tested patients, 6.4% were found to carry both *NLRP3* rs35829419 and *CARD8* rs2043211, correlating with a higher risk for RA [105,107], whereas an association with CD is less clear [142,143]. However, a combination of these *NLRP3* and *CARD8* SNPs conveys a protective effect against *Mycobacterium tuberculosis* infection of macrophages, accompanied by elevated levels of IL-1β in the blood serum of carriers. This suggests that an increase in pro-inflammatory NLRP3 activity due to these combined mutations could, on the one hand, worsen inflammatory diseases, but, on the other hand, could enhance the immune response against infections [130,134].

In conclusion, there is evidence for the contribution of several *CARD8* SNPs, partly in combination with *NLRP3* SNPs, to various pro-inflammatory diseases. However, more research is still required to confirm the functional relevance of *CARD8* SNPs in these diseases and elucidate the underlying molecular mechanisms [57].

## 8. Conclusions and Outlook

Although originally identified as an anti-inflammatory and anti-apoptotic protein [53,54,55], CARD8 is now also an established inflammasome sensor, which has been shown to detect viral protease activity [45,62]. The role of CARD8 as an inflammasome sensor is associated with its domain composition, since the FIIND and CARD highly resemble the carboxy-terminal effector fragment of NLRP1 [27,28]. Whereas CARDs can be found in numerous proteins involved in cell death and inflammation pathways, the FIIND occurs only in NLRP1 and CARD8. The FIIND is a constitutive autoproteolytic self-activation domain, resulting in a 1:1 expression ratio of the amino-terminal and carboxy-terminal polypeptides, ensuring the immediate inhibition of the carboxy-terminal effector fragment by the amino-terminal fragment after cleavage. In addition, the activity of the effector fragment is inhibited when bound to DPP8/9, representing a tightly regulated activation mechanism for these inflammasome sensors. However, unlike NLRP1, the CARD8 inflammasome does not require the adaptor protein ASC for caspase-1 recruitment and activation, and consequently does not induce ASC-speck formation, which is an activation hallmark of most other types of inflammasome sensors [16]. This suggests that CARD8 activates caspase-1 by homotypic CARD-CARD interactions. However, this stands in sharp contrast to publications which suggest an inhibition of caspase-1 by CARD8 [54,59]. The murine Nlrp1b can also form an inflammasome in the absence of ASC, sharing this property with CARD8, which is not expressed in mice [167]. Moreover, in contrast to NLRP1, CARD8 inflammasome activation mostly induces pyroptosis, rather than IL-1β and IL-18 secretion [57,64]. However, in some overexpression experiments, albeit less physiological, caspase-1 cleaves IL-1β in a CARD8-dependent manner. Furthermore, endothelial cells secrete IL-18 upon CARD8 activation [62,79]. More experiments are required to elucidate the molecular mechanisms underlying the regulation of caspase-1 activation induced by CARD8, as well as the factors influencing the apparent substrate specificity of the protease, which seems to depend on the recruiting inflammasome sensor. The FIIND of CARD8 acts not only as a proteolytic self-activation domain, but can also bind to, and inhibit, the activation of the inflammasome sensors NLRP3, NLRP1, and, possibly, AIM2 [89,90]. Therefore, a crosstalk, mostly mediated through direct interactions, seems to exist between CARD8 and the mentioned inflammasome sensors. Conversely, it is not known whether CARD8 activation can be inhibited by NLRP3, NLRP1, or AIM2. In endothelial cells, talabostat induces NLRP1 and CARD8 activation, while NLRP1 inhibits the ability of CARD8 to sense CVB3 [79,80]. In human keratinocytes, talabostat can only activate NLRP1, but not CARD8 [47]. However, when CARD8 T60 is overexpressed in NLRP1-knockout N/TERT-1 keratinocytes, it can be activated by talabostat, inducing the secretion of high amounts of IL-1β [43].

Genetic association studies, although inconclusive, suggest an involvement of *CARD8* in several inflammatory diseases. Therefore, pharmacological targeting of CARD8 might be a promising approach for the treatment of patients suffering from CARD8-associated diseases. CARD8 might have pro- or anti-inflammatory activity, depending on the diseases, the affected tissues, or cell types. A key for a better understanding of the contrasting pro- and anti-inflammatory functions of CARD8 might be the further analysis of the properties and expression patterns of its different isoforms. When CARD8 was initially described, it was believed that CARD8 T48 was the canonical isoform which binds and inhibits NLRP3, caspase-1/9, and NF-κB [53,54,55,59]. However, for virus-induced CARD8 inflammasome activation, the T60 isoform is required, since most of the known viral proteases target a disordered amino-terminal part only present in T60 [62,77]. The fact that CARD8 isoforms could also self-interact in a homo- and heterotypic manner with dominant-negative effects further complicates the situation [58,89]. Careful analysis of CARD8 isoform-specific mRNA and protein expression in different cell types, tissues, and diseases, combined with rescue experiments in knockout cells lacking CARD8 or other inflammasome sensors, might be an approach for the future elucidation of the molecular mechanisms underlying the multiple roles of CARD8 in inflammation and disease.
